# Paroxysmal Sympathetic Hyperactivity in a 73-Year-Old Female With Acute Myeloid Leukemia

**DOI:** 10.7759/cureus.16293

**Published:** 2021-07-10

**Authors:** Sravani Lokineni, Ekta Tirthani, Julia Wuest, Rania Ayyad

**Affiliations:** 1 Internal Medicine, Rochester Regional Health, Rochester, USA; 2 Oncology, Rochester Regional Health, Rochester, USA

**Keywords:** paroxysmal sympathetic hyperactivity, acute myeloid leukemia (aml), traumatic brain injury, sympathetic response, recurrence

## Abstract

Paroxysmal Sympathetic Hyperactivity (PSH) is a syndrome of recurrent exaggerated sympathetic responses in combination with motor features typically observed in the setting of traumatic brain injury and rarely seen without it. Here, we present a case of PSH in a 73-year-old female with acute myeloid leukemia (AML) without any brain injuries presenting with recurrent intermittent episodes of tachycardia, tachypnea, hypertension, fever, dystonia. These episodes resolved with clonidine and clonazepam thus confirming the diagnosis of PSH. PSH is an unusual and rare presentation in AML and not much literature has been reported.

## Introduction

Paroxysmal sympathetic hyperactivity is characterized by sympathetic storming with recurrent intermittent episodes of tachycardia, tachypnea, hypertension, fever, diaphoresis, dystonia. It is seen following multiple acquired insults to the brain like traumatic brain injury (TBI), anoxia, stroke, hemorrhage, infections. However, it is very rarely reported without any brain injury and particularly in acute myeloid leukemia. It is a challenging diagnosis of exclusion. With this report we highlight the importance of knowing specific etiologies of PSH, early diagnosis and treatment can significantly help in abating this neurologic emergency. 

## Case presentation

A 73-year-old female had a history of high-grade myelodysplastic syndrome with more than 10% blasts with new transformation to acute myeloid leukemia (AML) clinically and was started on chemotherapy with decitabine a day before admission. She presented with complaints of generalized weakness and a low-grade temperature of 38.2 Celsius. Three days after the admission, the patient experienced acute recurrent episodes of unresponsiveness accompanied by decreased heart rate to the 40s. This episode was followed by elevations in heart rate to 120-150 beats per minute, systolic blood pressure from 150-220 mmHg, tachypnea 20-40 breaths per minute, hyperthermia 38-39 degrees Celsius, along with some left-sided weakness and lethargy. Symptoms lasted between 20-40 min and occurred three to four times per day every 24 to 48 hours. During the examination, she was confused. Blood, fungal, and urine cultures were normal. Blood chemistries were unremarkable. 

Imaging studies with computed tomography (CT) without contrast and CT angiogram of the head were normal. Magnetic resonance imaging showed no evidence of new, acute/subacute cerebral or cerebellar infarct. Magnetic resonance venography showed patent venous sinuses. The patient was treated with levetiracetam because of the concern of seizures. Continuous video electroencephalogram did not show any evidence of seizural activity. The patient also underwent a lumbar puncture, which showed normal cerebrospinal fluid glucose, protein, cell count, and cytology. Cardiologic workup with an echocardiogram was unremarkable. Despite the seizure treatment on levetiracetam, the patient continued to have recurrent episodes of unresponsiveness along with tachycardia, hypertension, tachypnea, and hyperthermia. Due to lack of evidence and response to treatment, the patient was suspected of having paroxysmal sympathetic hyperactivity (PSH) with a score of 22 on a PSH assessment tool highly suggestive of the possibility. A trial of oral clonazepam 0.25 mg twice daily and clonidine 0.1 mg three times daily was given. Her episodes were abated, and no hemodynamic abnormalities were observed with the treatment, thereby confirming the diagnosis of PSH. On follow-up during the hospital course, she did not have any further episodes of PSH.

## Discussion

Paroxysmal sympathetic hyperactivity is defined as intermittent recurrent episodes of excessive sympathetic activity manifesting with tachycardia, tachypnea, hypertension, fever, diaphoresis, dystonia. It occurs suddenly but abates slowly. PSH can occur due to multiple causes of insults to the brain. It is reported [1] that the prevalence of PSH is 33% due to TBI compared to 6% by other causes. It is rarely seen in patients with acute myeloid leukemia (AML). There has only been a single case report [2] of a 53-year-old female with AML who presented with staring spells, jerky movement accompanied by hypertension, tachycardia, and tachypnea diagnosed with PSH and was successfully treated with intravenous morphine.

PSH [1, 2] is a complex syndrome consisting of sympathetic and motor features. It manifests with mainly six features, including tachycardia, tachypnea, hypertension, fever, diaphoresis, dystonic posturing. These clinical features can occur altogether or as a combination. In 2014, a clinical diagnostic tool [3] was proposed by an expert consensus group called PSH assessment measure (PSH-AM). It is scored based on clinical feature score (CFS) and diagnostic likelihood tool (DLT). Less than eight (< 8) is suggestive of less likely, > 17 as probable PSH, and 8-16 as possible. It should be noted that certain criteria may not be met due to variability of symptom appearance, and PSH may be misdiagnosed. It is important to exclude other possible diagnoses like seizures, severe sepsis, pulmonary embolism, intracranial hypertension, and herniation. These conditions can be detrimental and need to be investigated.

The pathophysiology behind PSH is not very clear. It is postulated [1, 4] the disconnection between descending inhibitory pathways and the sympathetic control center in the brain stem, diencephalon, and spinal cord leads to exaggerated sympathetic responses with minimal stimuli. Usually seen following diffuse brain injuries due to trauma, hemorrhage, infections. It is found that patients with deeper brain injuries in the periventricular white matter, corpus callosum, diencephalon, or brainstem are more likely to develop PSH than those with cortical and subcortical injuries [1].

Treatment [1, 2] of PSH usually includes general measures like reducing external stimuli, maintaining hydration, and pharmacological measures including abortive and preventive medications. Abortive medications include intravenous morphine, beta-blockers like propranolol, dexmedetomidine. Preventative medications include gabapentin, clonidine, benzodiazepines and beta-blockers. Evidence for any particular treatment is limited. In a recent case of refractory PSH in a young female with anoxic brain injury following cardiac arrest, it was noted that enteral baclofen [5] prevented the sustained symptoms and reduced the hospitalization stay. It was also found that trazodone [6] was effective in reducing the PSH symptoms in a 49-year-old woman following a left temporal subcortical hemorrhage.

## Conclusions

PSH is mostly reported following TBI. This is a unique case in which PSH is associated with acute myeloid leukemia. It is not understood if AML is causative or correlated. We need more studies to understand its pathophysiology which can help in early diagnosis and treatment. Delayed diagnosis can lead to secondary complications like dehydration, malnutrition, weight loss, muscle contractures, and prolonged intensive care stay.
